# Assessing Agreement between Radiomic Features Computed for Multiple CT Imaging Settings

**DOI:** 10.1371/journal.pone.0166550

**Published:** 2016-12-29

**Authors:** Lin Lu, Ross C. Ehmke, Lawrence H. Schwartz, Binsheng Zhao

**Affiliations:** 1 Department of Radiology, Columbia University Medical Center, New York, NY, United States of America; 2 Department of Medicine, Columbia University Medical Center, New York, NY, United States of America; Institute of Automation Chinese Academy of Sciences, CHINA

## Abstract

**Objectives:**

Radiomics utilizes quantitative image features (QIFs) to characterize tumor phenotype. In practice, radiological images are obtained from different vendors’ equipment using various imaging acquisition settings. Our objective was to assess the inter-setting agreement of QIFs computed from CT images by varying two parameters, slice thickness and reconstruction algorithm.

**Materials and Methods:**

CT images from an IRB-approved/HIPAA-compliant study assessing thirty-two lung cancer patients were included for the analysis. Each scan’s raw data were reconstructed into six imaging series using combinations of two reconstruction algorithms (Lung[L] and Standard[S]) and three slice thicknesses (1.25mm, 2.5mm and 5mm), i.e., 1.25L, 1.25S, 2.5L, 2.5S, 5L and 5S. For each imaging-setting, 89 well-defined QIFs were computed for each of the 32 tumors (one tumor per patient). The six settings led to 15 inter-setting comparisons (combinatorial pairs). To reduce QIF redundancy, hierarchical clustering was done. Concordance correlation coefficients (CCCs) were used to assess inter-setting agreement of the non-redundant feature groups. The CCC of each group was assessed by averaging CCCs of QIFs in the group.

**Results:**

Twenty-three non-redundant feature groups were created. Across all feature groups, the best inter-setting agreements (CCCs>0.8) were 1.25S vs 2.5S, 1.25L vs 2.5L, and 2.5S vs 5S; the worst (CCCs<0.51) belonged to 1.25L vs 5S and 2.5L vs 5S. Eight of the feature groups related to size, shape, and coarse texture had an average CCC>0.8 across all imaging settings.

**Conclusions:**

Varying degrees of inter-setting disagreements of QIFs exist when features are computed from CT images reconstructed using different algorithms and slice thicknesses. Our findings highlight the importance of harmonizing imaging acquisition for obtaining consistent QIFs to study tumor imaging phonotype.

## Introduction

Radiomics seeks to use a large number of quantitative image features (QIFs) extracted from non-invasive, routinely acquired radiologic images to characterize tumor phenotypes [1–4]. Radiomics has shown promise in improving cancer diagnosis and prognosis in several tumor types including lung [5–10], brain [11,12], liver [13], kidney [14] and esophageal [15] cancers. While the field of radiomics continues to evolve, a potential limitation in image analysis is the heterogeneous imaging acquisition parameters being used, i.e., there exists a wide range of imaging equipment, acquisition techniques and reconstruction parameters in clinical practice and even in clinical trials. Thus it is important to determine how different imaging acquisition parameters affect computed values of QIFs. Greater understanding of the effect of imaging acquisition choices on radiomic feature variability will lead to increased confidence in the validity of radiomic analyses, inform the standardization of imaging parameters, and enhance generalization and applicability of findings in the rapidly growing field of radiomics.

Quantitative image features in radiomics are computed from digital images. They have both spatial resolution (voxel size) and gray-level/density resolution (density bin size) which are determined by imaging acquisition techniques and parameters. To date, our knowledge of the reliability of QIFs is limited to studies of CT and PET test-retest reproducibility [16–19], intra- and inter-observer variability [20,21], segmentation method-induced variability [22,23], variation due to CT acquisition parameters (phantom study) [24], and effects of different CT scanners [25]. There was no in vivo study that assessed how CT imaging acquisition parameters such as slice thickness and reconstruction algorithm affect the computations of QIFs, until the recently published same-day repeat CT study [26]. In that study, Zhao *et al*. focused on investigating the effect of re-imaging on reproducibility of QIFs under a same imaging setting condition between two repeat scans[26]. In the present study, an urgent unmet need is to assess the agreement between QIFs computed at different imaging settings covering the range of CT imaging reconstruction parameters that are widely used in current clinical practice and oncology clinical trials.

## Materials and Methods

### CT Imaging Data

A CT imaging data set of 32 non-small cell lung cancer (NSCLC) patients was collected from a previous institutional review board (IRB) approved and Health Insurance Portability and Accountability Act (HIPAA) compliant repeat CT study (ClinicalTrials.gov identifier NCT00579852). In the original study, 32 lung cancer patients underwent two repeat CT scans within 15 minutes. GE scanners were used and the imaging protocol is provided in Table 1. To date, only the repeat imaging series of 1.25L in the protocol was primarily used to study the reproducibility of tumor volume and diameter [21] and became publicly available through the National Cancer Institute’s Reference Image Database to Evaluate Therapy Response (RIDER) project [27]. Since then, researchers around the world have used this publicly accessible data set to validate their computational methods and hypotheses [1–3,16,18,19].

**Table 1:** Imaging protocol of the 32 CT scans.

Scanning parameters	GE LightSpeed 16 (28/32)	GE VCT (4/32)
**Tube voltage**	120 kVp	120 kVp
**Tube current**	299–441 mA	298–351 mA
**Collimator configuration**	16 x 1.25 mm	64 x 0.63 mm
**Pitch**	1.375	0.984
**In-plane resolution**	0.51x0.51~0.90x0.90	0.50x0.50~0.87x0.87
**slice thickness**	1.25, 2.5 and 5 mm	1.25, 2.5 and 5 mm
**Reconstruction algorithm**	Lung (L) and Standard (S)	Lung (L) and Standard (S)
**Contrast**	No	No

**Fig 1:**
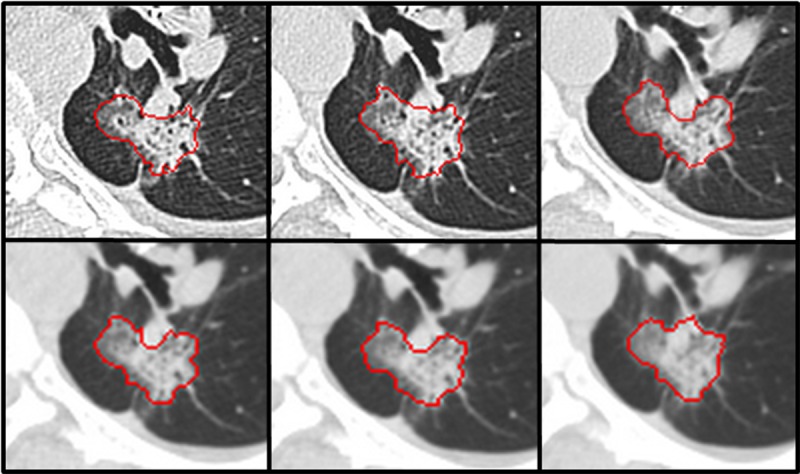
An example of a lung lesion on the images reconstructed at the six acquisition settings. The settings of 1.25L, 2.5L and 5L line at the upper row. The settings of 1.25S, 2.5S and 5S line at the bottom row. For example, the name of setting 1.25L means the imaging setting of slice thickness of 1.25mm and Lung reconstruction. Arrow heads in the image of setting 1.25L indicate the tumor lesion.

In the present study, the raw data of each patient’s first CT scan were reconstructed into six image series: combinations of three slice thicknesses (1.25mm, 2.5mm and 5mm) and two reconstruction algorithms [lung (L) and standard (S)], and used to study the agreement between radiomic features computed for multiple imaging parameters. Fig 1A–1F show an example of a lung lesion imaged on the six series of 1.25L, 2.5L, 5L, 1.25S, 2.5S and 5S.

### Lung Lesion Segmentation

Prior to feature extraction, a lesion needs to be separated from its surrounding background. In this study, lesion segmentation was performed using a semi-automated algorithm based on an approach combining the watershed and active contours [28]. Three radiologists (with 11, 10 and 25 years’ experience of interpreting oncologic CT images, respectively) were assigned to independently segment the 32 lesions (one lesion per patient) on all six image series at multiple sessions. To reduce the effect of variations in the radiologist’s memory, each segmentation session contained only one series and each session was separated by three weeks. Each segmentation result was allowed to be edited by the radiologist if any computer-generated result was considered unsatisfactory. The final segmentation used for feature extraction was the consensual results of at least 2 of the 3 radiologists, i.e., each lesion voxel had to be contained in the lesion contours delineated by at least 2 of the 3 radiologists. The inter-rater agreement among radiologists and final results were provided in the S1 File.

### Quantitative Image Feature Extraction

From each segmented lesion, we extracted 89 well-defined QIFs. These features are used to quantify tumor size (e.g., volume), shape (e.g., eccentricity, compactness), boundary shape (e.g., shape index), sharpness (e.g., sigmoid slope), density distributions without spatial information (e.g., histogram-derived density statistics of mean and standard deviation), and density distributions with spatial information, also known as texture patterns (e.g., fine texture features such as wavelet features derived at lower decomposition levels; coarse texture features such as Loge computed from images smoothed using a larger Gaussian kernel). The definitions of the 89 QIFs along with their relevant references can be found in the S2 File, and the numerical values of the 89 QIFs can be found in S1 Table.

In the implementation, all CT images were resampled into isotropic data with a voxel spacing of 0.5 × 0.5 × 0.5 *mm*^3^ by using tri-linear interpolation before computing the features. This is the only preprocessing done to the data. No additional optimization was performed to the 89 QIFs. We used the same feature parameters across all imaging settings in this study.

### Feature Redundancy Reduction

There was potential redundancy in the 89 QIFs. For example, the diameter and volume are both features describing the tumor size. To minimize over weighting on redundant QIFs, we used a hierarchical clustering method [29,30] to identify redundant features prior to the assessment of inter-setting agreement. The process of redundancy reduction consisted of two steps. Firstly, we built up a hierarchical cluster tree of the 89 QIFs based on similarity measurement. Secondly, we explored the relationship between similarity threshold and the number of non-redundant clusters. After building the similarity clusters, the redundant/correlated QIFs within each cluster were combined into a new QIF. The value of a new QIF was the average of the value of the QIFs in the cluster.

**Fig 2:**
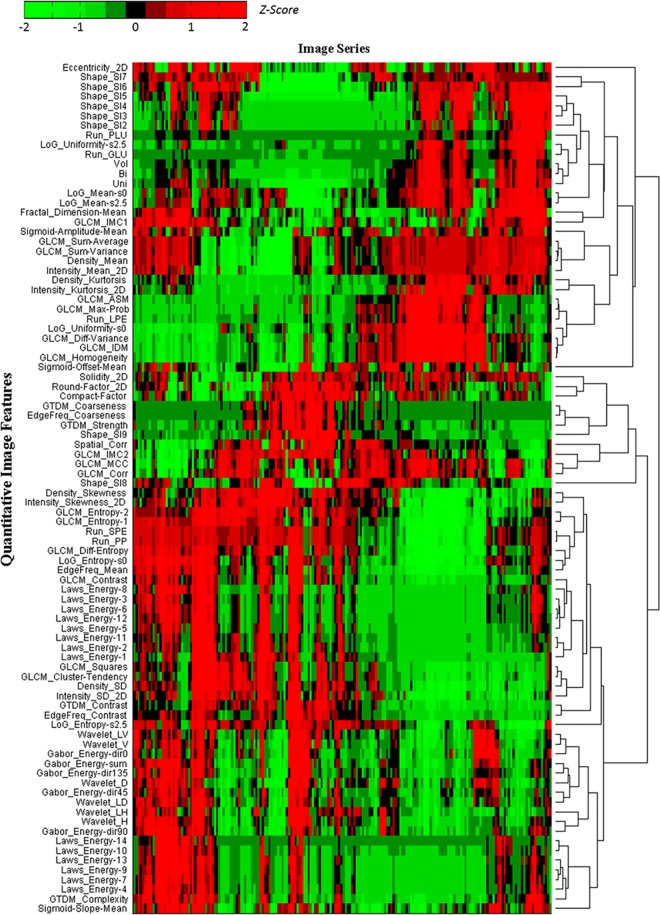
The hierarchical cluster tree of the 89 QIFs across 32 tumors at six imaging settings.

At the first step, a hierarchical cluster tree for the 89 QIFs was generated by adopting the hierarchical clustering method proposed in the literature [29]. The hierarchical clustering method was intrinsically an iteration process based on the similarity among QIFs. At each iteration of the clustering method, two of the most similar clusters were combined into one cluster and then put back as one cluster for the next iteration. A cluster node in the hierarchical cluster tree could be one individual QIF or several QIFs. The generated cluster tree for the 89 QIFs was presented in Fig 2 at the Result section.

It is noted that, once the hierarchical cluster tree was built, z-score transformation was applied to standardize all the QIF values, because different QIFs would have different value ranges. For instance, in our study, the ‘volume’ feature has a range of 2 × 10^3^∼1.5 × 10^5^ mm^3^, while the ‘mean intensity’ feature has a range of -520~60 *Hounsfield units*. Z-score transformation was defined as below, aiming to center QIF value to have mean 0 and standard deviation 1.

$$z=\frac{\left(x-\mu \right)}{\sigma }$$ (1)

Where *μ* was the mean value of QIF and *σ* was the standard deviation of QIF.

After applying z-score transformation, different QIFs would be scaled to a standardized value range, usually from -3 to 3 (corresponding to the original range of (*μ*−3*σ*)∼(*μ*+3*σ*)). The overall hierarchical clustering process was implemented on the platform of Matlab R2012b (The Mathworks, Natick, MA). The similarity between two clusters was measured by unweighted average Spearman’s rank correlation coefficient [30].

After building up a hierarchical cluster tree, we plotted out the relationship curve between the similarity threshold and the number of clusters. It is obvious that, smaller similarity thresholds resulted in larger number of clusters. Large number of clusters means the existing of much redundancy. Yet, too small number of clusters might bias the further agreement analysis. Therefore, a tradeoff similarity threshold was to be selected via exploring the relationship curve. Details and experimental results could be found in the ‘Result’ section.

### Inter-Setting Agreement Analysis

Concordance correlation coefficients (CCCs) [31] were used to assess inter-setting agreement of QIFs. The six imaging settings resulted in $C_{6}^{2}=15$ pair-wise comparisons.

Let *x* and *y* be the compared non-redundant QIFs calculated from a pair of inter-setting comparisons. The definition of CCC is as follows:

$$ccc=\frac{2\rho \sigma_{x}\sigma_{y}}{\sigma_{x}^{2}+\sigma_{y}^{2}+{\left(\mu_{x}-\mu_{y}\right)}^{2}}$$ (2)

where *μ*_*x*_ and *μ*_*y*_ are the mean values of *x* and *y*. *σ*_*x*_ and *σ*_*y*_ are the variances of *x* and *y*. *ρ* is the correlation coefficient between *x* and *y*. CCC evaluates a deviation from the 45° identity line between two compared data sets with its value ranging from -1 to 1.

## Results

### Non-Redundant QIF Groups

The hierarchical cluster tree of the 89 QIFs across 32 tumors at six imaging settings is presented in Fig 2. Noticeably, in the hierarchical cluster tree, several QIFs such as the Laws'_energy features have very similar values (z-scores), indicating the redundancy of these features. Similarity thresholds were used to combine such similar QIFs into one cluster.

**Fig 3:**
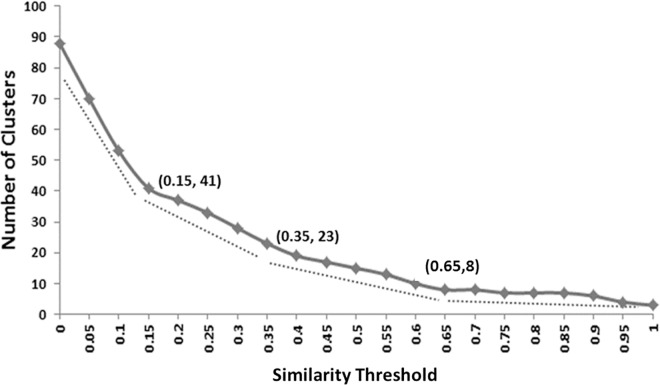
The relationship between similarity threshold and the number of non-redundant clusters. Dotted lines show the descending trend of the curve. **Similarity** threshold = 0.35 was selected, generating 23 non-redundant QIF groups for agreement analysis.

Fig 3 shows the relationship between the similarity threshold and the number of clusters. As expected, higher setting of similarity threshold results in smaller number of non-redundant clusters. As can be seen in Fig 3, there are four descending trends (four dotted line segments) on the curve. This led to three candidate thresholds, 0.15, 0.35 and 0.65. The number of clusters dropped quickly between 0 and 0.15. At the threshold of 0.15, the 89 QIFs collapsed into 41 clusters, meaning that about half of the 89 QIFs were redundant (similar). In contrast, the number of clusters became stable when thresholds exceeded 0.65. So, in this study, the similarity threshold 0.35 was selected a tradeoff between the thresholds of 0.15 and 0.65.

### Inter-Setting Agreements of QIFs

**Fig 4:**
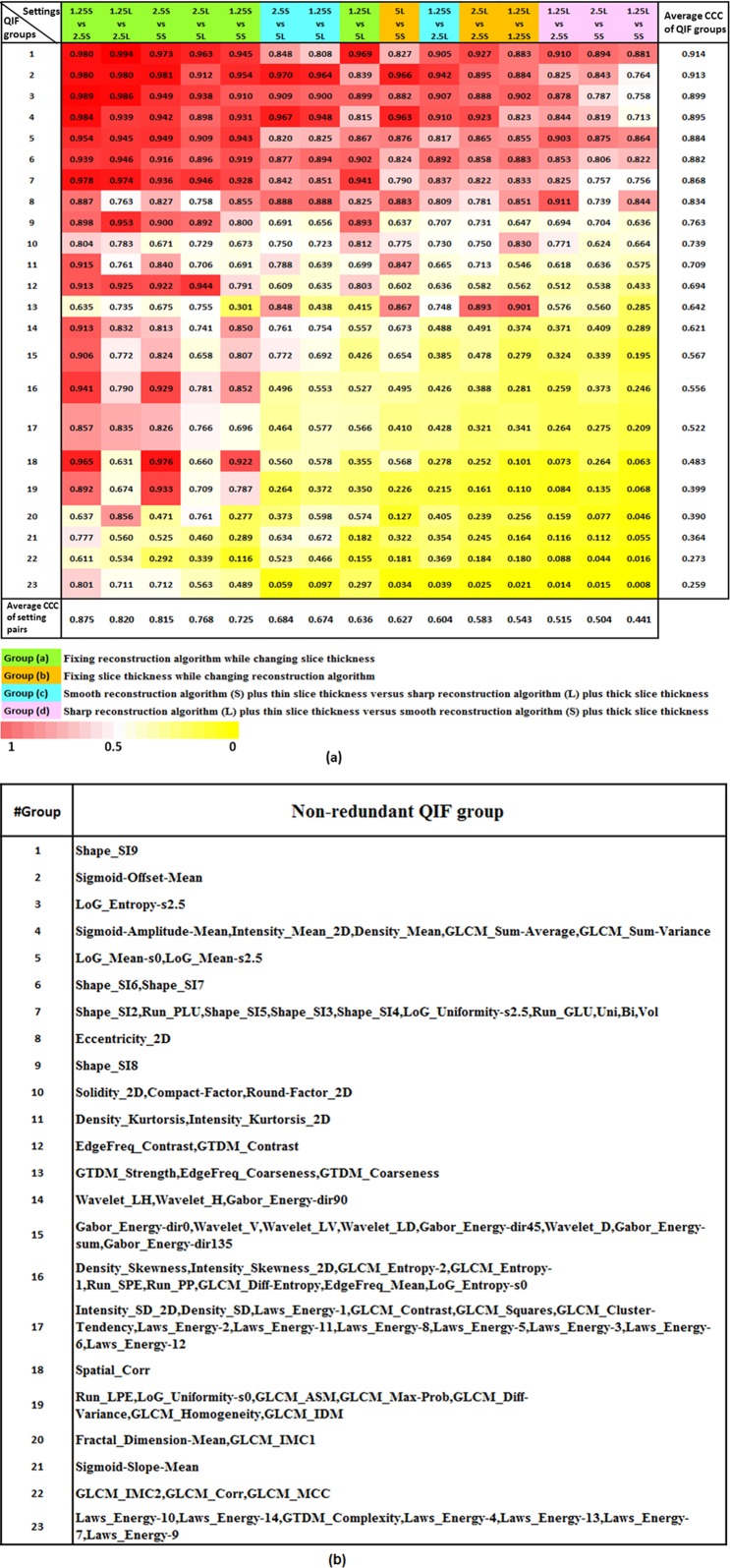
(a) The CCCs of non-redundant QIF groups under the 15 inter-setting comparisons. Columns are arranged in descending order according to the average CCC of the inter-setting comparisons. Rows are arranged in descending order according to average CCCs of Non-redundant QIF Groups. (b) The clustered 23 non-redundant feature groups and the features in each group.

The CCCs of the 89 QIFs across all of the 15 inter-setting comparisons were calculated (See Supplementary Information for details). The CCC for each non-redundant QIF group was attained by averaging all CCCs of the QIFs in the group. Fig 4 shows the CCCs of the 23 non-redundant QIF groups across the 15 inter-setting comparisons. To compare agreements with one or two setting changes, we categorized the 15 inter-setting comparisons into the following four sub comparison groups: i) fixing reconstruction algorithm while changing slice thickness, ii) fixing slice thickness while changing reconstruction algorithm, iii) combination of Standard (smooth, S) reconstruction algorithm and ‘thinner’ slice thickness versus Lung (sharp, L) reconstruction algorithm and ‘thicker’ slice thickness, and iv) combination of Lung reconstruction algorithm and ‘thinner’ slice thickness versus Standard reconstruction algorithm and ‘thicker’ slice thickness. The definition of the ‘thinner’ and the ‘thicker’ in the combinations were based on the rule that a ‘thinner’ slice thickness was always smaller than a ‘thicker’ slice thickness and vice versa.

In Fig 4A, we arranged the 15 inter-setting comparisons (from left to right) in descending order of the average CCCs of the 23 non-redundant feature groups and, for better visual comparison, colored the groups a), b), c) and d) with green, orange, blue and purple respectively. Three inter-setting comparisons, i.e., 1.25S vs 2.5S, 1.25L vs 2.5L and 2.5S vs 5S, showed the average CCCs of all 23 groups greater than 0.8 (bottom row in Fig 4A). Overall, the agreement trend of the four comparison groups was a) > c) > b) > d). The top rank of group a) indicated that, changing slice thickness alone yielded the best agreement levels, especially when the range of slice thickness was confined to 1.25mm and 2.5mm. The ranks of groups a) and b) showed that the agreement levels of changing reconstruction algorithm were worse than those of changing slice thickness. By comparing groups b) and c), an interesting finding was that changing two parameters could have higher agreement levels than changing one parameter (i.e., reconstruction algorithm), under the condition of Standard reconstruction algorithm and ‘thinner’ slice thickness versus Lung reconstruction algorithm and ‘thicker’ slice thickness. Yet, under these converse conditions of Lung reconstruction algorithm and ‘thinner’ slice thickness versus Standard reconstruction algorithm and ‘thicker’ slice thickness, the worst agreement levels occurred with group d). The worst comparison was that of the Lung reconstruction algorithm and the thinnest slice thickness of 1.25mm versus Standard reconstruction algorithm and the thickest slice thickness of 5mm, with an average CCC smaller than 0.5.

Also in Fig 4A, we arranged the non-redundant feature groups (from top to bottom) in descending order of the average CCCs of each feature group across the 15 inter-setting comparisons. The top eight QIF groups showed acceptable agreement levels with the average CCC values (across all of the 15 inter-setting comparisons) > 0.8. The eight QIF groups consisted of the features related to tumor size, mean density, coarse boundary morphology (e.g., Shape Index 9) and coarse textures (e.g., LoG_Entropy_s2.5). In contrast, twelve QIF groups characterizing tumor high-order density distributions (e.g., Skewness), boundary sharpness (Sigmoid Slope) and fine textures (e.g., GLCM) all showed low agreement levels: an average CCC < 0.7. The poorest performing QIF group was the Laws'_Energy features with an average CCC as low as 0.259. The low agreement levels of boundary sharpness and fine texture QIFs suggested that detailed information of tumors were more easily affected by the change of image acquisition parameters.

## Discussion

The use of radiological image features to quantitatively characterize tumor phenotypes is an area of rapidly growing research. To date, most of the published radiomics work has been based on retrospective studies in which imaging data were acquired without consideration for the suitability for computing complex and/or fine image features such as texture futures. Despite the availability of many manufacturer’s models and versions of CT scanners, differing imaging acquisition techniques and reconstruction parameters that have been used in clinical care, little is known about how such variations in imaging acquisition methods affect quantitative features. We conducted the present study to explore this knowledge gap.

In this study, we chose 89 comprehensive, commonly used QIFs and categorized them into non-redundant feature groups. We then analyzed the agreements of these features when computed from CT images reconstructed using different slice thicknesses and reconstruction algorithms. The slice thicknesses studied ranged from 1.25mm to 5mm and the reconstruction algorithms included both smooth and sharp algorithms. These acquisition parameters are representative of the CT imaging variety used in current oncology practices and clinical trials.

We found that QIF inter-setting disagreements existed and varied with the imaging parameters and the types of the QIFs. Across all of the non-redundant feature groups, maintaining the same reconstruction algorithm while calculating QIFs at 1.25mm and 2.5mm slice thickness images showed the best agreement (CCCs>0.8), indicating the potential interchangeable use of these two imaging settings. The worst combination among all imaging settings belonged to 1.25L vs 5S (CCCs<0.5), i.e., the thinnest slice (1.25mm) reconstructed using the Lung (sharp) algorithm versus the thickest slice (5mm) reconstructed using the Standard (smooth) algorithm. Because thicker slices introduce larger partial volume artifacts and thus blur and smooth images more than thinner slices, such smoothing effects caused by using thicker slices are similar to that brought in by using smooth reconstruction algorithms. This also explains why combining thicker slices with sharper reconstruction algorithms have similar effects as combining thinner slices with smoother reconstruction algorithms in the computation of QIFs (CCCs>0.6).

Our findings also show that different types of QIFs can be affected to varying degrees by the imaging settings. The QIFs characterizing tumor size, boundary morphology, low order density statistics and coarse textures were less sensitive to the imaging setting parameters than the QIFs characterizing tumor boundary sharpness, high-order density statistics and fine textures. This occurred because the former feature groups mainly relied on the segmented tumor boundaries and/or characterized low frequency change components, whereas the latter feature groups are characterized by high frequency change components.

Our study has several limitations. First, we only studied the effects of two imaging acquisition variables, slice thickness and reconstruction algorithm, on the extraction of QIFs. There are other CT acquisition variables, such as kVp, mA and pitch that would also affect image quality and thus potentially affect QIFs. Nevertheless, slice thickness and reconstruction algorithm are two major variables. Second, the patient CT images were all collected on GE scanners. Due to the nature of human study, we were only able to study the imaging settings provided by GE scanners. But the conclusions drawn from our study should be applicable to the comparable imaging settings from other CT vendors’ scanners. Phantom studies are anticipated to be designed to systematically explore QIF differences caused by machine, dose, and so on. Third, our results were obtained using lung cancer images. Effects of CT imaging acquisition on QIFs may vary with different tumors residing at different anatomic sites.

## Conclusions

Our study reveals that variations exist in the values of QIFs when extracted from CT images reconstructed using different acquisition parameters. This should raise awareness of the necessity of harmonizing imaging acquisition techniques not only for CT, but also for PET and MRI studies, to accelerate the identification of optimal quantitative image features and thus imaging phenotypes in radiomics research.

## Supporting Information

S1 File The inter-rater agreement on segmentation among radiologists and final results. (DOCX)

S2 File The definitions of quantitative image features. (DOCX)

S1 Table The numerical values of the 89 extracted quantitative image featrues. (XLSX)
