# Supplementary material for: Assessing Agreement between Radiomic Features Computed for Multiple CT Imaging Settings
Source: PLoS One. 2016 Dec 29;11(12):e0166550. doi: 10.1371/journal.pone.0166550 (PMC5199063; doi:10.1371/journal.pone.0166550)
Supplement: S1 File — (DOCX) [file pone.0166550.s001.docx]

**S1 File. The inter-rater agreement on segmentation among radiologists and final results.**

***The inter-rater agreement among radiologists and final results***

In this study, three radiologists (R1, R2 and R3, with 13, 12 and 25 year experience of interpreting oncologic CT images) were assigned to segment the 32 lesions independently at multiple sessions (one session for one imaging settings). The software they used had been well validated [28]. During the delineation of tumor, computer result was allowed to be edited by radiologists. This is the way a tumor segmentation algorithm is being used in research practice and clinical trail. The frequency of manual editing was not recorded.

The average dice similarities between two of the three radiologists were 0.82, 0.87 and 0.85, respectively. The dice similarity was defined as below,

$$Dice=\frac{2\left| X\cap Y \right|}{\left| X \right|+\left| Y \right|}$$

Where $X$ and $Y$ were the volumes of two contours to be compared.

To attain ‘Gold standard’, we took the consensual results of at least 2 of the 3 radiologists, i.e., each lesion voxel had to be contained in the lesion contours delineated by at least 2 of the 3 radiologists. The average dice similarity between the final consensual results and the three radiologists were 0.92, 0.89 and 0.95, respectively. We can see that, the final consensual results were very close to those three radiologists. The dice similarity results were present below.

| **Table 1 The dice similarity among radiologists and final results** |  | | |  | | | |  |
| --- | --- | --- | --- | --- | --- | --- | --- | --- |
|  |  |  |  | |  |  |  | |
| \| **Dice Similarity** \| **Inter-Radiologists** \| \| \| **Consensual Results**  **vs Individual Radiologists** \| \| \| \| --- \| --- \| --- \| --- \| --- \| --- \| --- \| \| **R1vs R2** \| **R1vs R3** \| **R2vs R3** \| **Consensus vs R1** \| **Consensus vs R2** \| **Consensus vs R3** \| \| **Lesion1** \| **0.95** \| **0.94** \| **0.95** \| **0.97** \| **0.98** \| **0.97** \| \| **Lesion2** \| **0.72** \| **0.75** \| **0.86** \| **0.82** \| **0.90** \| **0.93** \| \| **Lesion3** \| **0.84** \| **0.85** \| **0.81** \| **0.94** \| **0.90** \| **0.90** \| \| **Lesion4** \| **0.95** \| **0.97** \| **0.95** \| **0.98** \| **0.97** \| **0.98** \| \| **Lesion5** \| **0.69** \| **0.77** \| **0.73** \| **0.86** \| **0.82** \| **0.89** \| \| **Lesion6** \| **0.98** \| **1.00** \| **0.99** \| **1.00** \| **0.99** \| **1.00** \| \| **Lesion7** \| **0.89** \| **0.97** \| **0.91** \| **0.98** \| **0.91** \| **0.99** \| \| **Lesion8** \| **0.92** \| **0.93** \| **0.92** \| **0.97** \| **0.95** \| **0.96** \| \| **Lesion9** \| **0.58** \| **0.60** \| **0.87** \| **0.66** \| **0.92** \| **0.93** \| \| **Lesion10** \| **0.54** \| **0.56** \| **0.84** \| **0.63** \| **0.89** \| **0.92** \| \| **Lesion11** \| **0.95** \| **0.98** \| **0.95** \| **0.99** \| **0.96** \| **0.99** \| \| **Lesion12** \| **0.85** \| **0.90** \| **0.89** \| **0.94** \| **0.92** \| **0.96** \| \| **Lesion13** \| **0.86** \| **0.92** \| **0.87** \| **0.96** \| **0.90** \| **0.96** \| \| **Lesion14** \| **0.87** \| **0.85** \| **0.89** \| **0.92** \| **0.95** \| **0.93** \| \| **Lesion15** \| **0.86** \| **0.88** \| **0.84** \| **0.95** \| **0.91** \| **0.93** \| \| **Lesion16** \| **0.86** \| **0.97** \| **0.84** \| **1.00** \| **0.87** \| **0.97** \| \| **Lesion17** \| **0.75** \| **0.78** \| **0.85** \| **0.85** \| **0.90** \| **0.93** \| \| **Lesion18** \| **0.90** \| **0.94** \| **0.90** \| **0.97** \| **0.93** \| **0.97** \| \| **Lesion19** \| **0.87** \| **0.94** \| **0.88** \| **0.96** \| **0.90** \| **0.97** \| \| **Lesion20** \| **0.91** \| **0.94** \| **0.91** \| **0.97** \| **0.94** \| **0.97** \| \| **Lesion21** \| **0.29** \| **0.72** \| **0.37** \| **0.80** \| **0.40** \| **0.94** \| \| **Lesion22** \| **0.93** \| **0.96** \| **0.94** \| **0.98** \| **0.95** \| **0.98** \| \| **Lesion23** \| **0.76** \| **0.90** \| **0.73** \| **0.96** \| **0.79** \| **0.94** \| \| **Lesion24** \| **0.86** \| **0.96** \| **0.87** \| **0.98** \| **0.88** \| **0.98** \| \| **Lesion25** \| **0.87** \| **0.88** \| **0.89** \| **0.93** \| **0.94** \| **0.95** \| \| **Lesion26** \| **0.93** \| **0.94** \| **0.94** \| **0.96** \| **0.97** \| **0.97** \| \| **Lesion27** \| **0.68** \| **0.74** \| **0.78** \| **0.83** \| **0.85** \| **0.91** \| \| **Lesion28** \| **0.68** \| **0.87** \| **0.69** \| **0.94** \| **0.74** \| **0.92** \| \| **Lesion29** \| **0.81** \| **0.82** \| **0.83** \| **0.91** \| **0.90** \| **0.90** \| \| **Lesion30** \| **0.92** \| **0.91** \| **0.90** \| **0.96** \| **0.96** \| **0.94** \| \| **Lesion31** \| **0.79** \| **0.85** \| **0.74** \| **0.95** \| **0.84** \| **0.89** \| \| **Lesion32** \| **0.82** \| **0.81** \| **0.77** \| **0.94** \| **0.89** \| **0.86** \| \| **Average** \| **0.82** \| **0.87** \| **0.85** \| **0.92** \| **0.89** \| **0.95** \| |  |  |  | |  |  |  | |
|  |  |  |  | |  |  |  | |
|  |  |  |  | |  |  |  | |
|  |  |  |  | |  |  |  | |
|  |  |  |  | |  |  |  | |
|  |  |  |  | |  |  |  | |
|  |  |  |  | |  |  |  | |
|  |  |  |  | |  |  |  | |
|  |  |  |  | |  |  |  | |
|  |  |  |  | |  |  |  | |
|  |  |  |  | |  |  |  | |
|  |  |  |  | |  |  |  | |
|  |  |  |  | |  |  |  | |
|  |  |  |  | |  |  |  | |
|  |  |  |  | |  |  |  | |
|  |  |  |  | |  |  |  | |
|  |  |  |  | |  |  |  | |
|  |  |  |  | |  |  |  | |
|  |  |  |  | |  |  |  | |
|  |  |  |  | |  |  |  | |
|  |  |  |  | |  |  |  | |
|  |  |  |  | |  |  |  | |
|  |  |  |  | |  |  |  | |
|  |  |  |  | |  |  |  | |
|  |  |  |  | |  |  |  | |
|  |  |  |  | |  |  |  | |
|  |  |  |  | |  |  |  | |
|  |  |  |  | |  |  |  | |
|  |  |  |  | |  |  |  | |
|  |  |  |  | |  |  |  | |
|  |  |  |  | |  |  |  | |
|  |  |  |  | |  |  |  | |
|  |  |  |  | |  |  |  | |
